# West Nile Virus, Venezuela

**DOI:** 10.3201/eid1304.061383

**Published:** 2007-04

**Authors:** Irene Bosch, Flor Herrera, Juan-Carlos Navarro, Miguel Lentino, Alan Dupuis, Joseph Maffei, Matthew Jones, Ernesto Fernández, Nelson Perez, Jorge Pérez-Emán, Anthony Érico Guimarães, Roberto Barrera, Nereida Valero, Johanny Ruiz, Glenda Velásquez, Juán Martinez, Guillermo Comach, Nicholas Komar, Andrew Spielman, Laura Kramer

**Affiliations:** * University of Massachusetts Medical School, Worcester, Massachusetts, USA; † Universidad de Carabobo Biomed, Maracay, Venezuela; ‡ Universidad Central de Venezuela, Caracas, Venezuela; § Coleccion Ornitologica Phelps, Caracas, Venezuela; ¶ New York State Department of Health, Albany, New York, USA; # State University of New York at Albany, Albany, New York, USA; ** Universidad Central de Venezuela, Maracay, Venezuela; †† Instituto Nacional de Investigaciones Agrícolas, Maracay, Venezuela; ‡‡ Instituto Oswaldo Cruz, Rio de Janeiro, Brazil; §§ Centers for Disease Control and Prevention, San Juan, Puerto Rico, USA; ¶¶ Universidad del Zulia, Maracaibo, Venezuela; ## Ministerio de Salud Insalud, Carabobo, Venezuela; *** Centers for Disease Control and Prevention, Fort Collins, Colorado, USA; ††† ^1^Harvard School of Public Health, Boston, Massachusetts, USA; 1 Deceased.

**Keywords:** West Nile virus, Venezuela, Saint Louis encephalitis virus, Ilheus virus, ELISA, PRNT, letter

**To the Editor:** West Nile virus (WNV; genus *Flavivirus*; family *Flaviviridae*) has been perpetuating in North America since 1999 (*1*). However, its status as a self-perpetuating pathogen in South America remains uncertain. Infected horses and birds have been reported in various Caribbean Islands, Mexico, and northern Central America (*2*,*3*). In South America, isolated reports of infected dead-end hosts (horses) have come from northern Colombia and Argentina but they lack evidence for infection in avian amplifying hosts (*4*,*5*). We report serologic evidence of establishment of WNV in South America.

Serum samples from birds and horses from 33 locations in Venezuela (Appendix Table) were screened for immunoglobulin G (IgG) antibodies against WNV antigen by ELISA (*6*) and confirmed by plaque reduction neutralization test (PRNT) as previously described (*7*). The flavivirus generating the IgG response was identified by using the following criteria: 90% inhibition of virus in serum diluted at least 1:40 and 4-fold greater neutralizing antibody titer compared with closely related flaviviruses. IgG antibody against flavivirus was detected by ELISA in 14 of 576 resident birds, including 5 *Turdus leucomelas*, 3 *Gallus gallus* (captive), 2 *Campylorhamphus trochilirostris*, and 1 each of *Elaenia flavogaster*, *Coereba flaveola*, *Thraupis palmarum*, and *Anisognathus flavinucha*.

WNV was confirmed as the etiologic agent of infection in 5 adult birds (3 *T*. *leucomelas* [pale-breasted thrush], 1 *C*. *flaveola* [bananaquit], and 1 *G*. *gallus* [domestic chicken] with the earliest collection date in February 2006); virus neutralization titers ranged from 80 to 320. One serum sample cross-reacted with other flaviviruses tested, with equivalent titers to WNV, Saint Louis encephalitis virus (SLEV), and Ilheus virus (ILHV) and was thus considered infected with an undetermined flavivirus. Seven serum samples were negative (antibody titers <20), and 1 sample was not tested because of insufficient sample volume.

Antibody against flavivirus was detected by ELISA in 141 of 791 horses, and 34 (4.3%) were confirmed positive for WNV infection by PRNT; viral titers ≥640 occurred in half of these horses. The earliest collection date for a WNV-positive horse was February 2004 and the most recent was May 2006. Specific WNV-reactive equine serum samples were distributed in valley regions (prevalence 1.3%), savannah grasslands (2.4%), the western region of Zulia (0.4%) and the Central Lake Basin (0.3%). A total of 46 (5.8%) equine serum samples were positive for neutralizing antibody to SLEV, and 8 (1.0%) samples were positive for neutralizing antibodies to ILHV. Forty-nine samples neutralized at least 2 of the 3 viruses and were classified as undetermined flaviviruses. Serum samples from 2 horses were negative in neutralization assays; 2 others were not tested because of insufficient sample volume.

WNV-infected resident birds, rather than an importation event, are the basis of establishment of WNV in South America. We hypothesize that ornithophilic mosquitoes (such as some *Culex* spp.), which are present in the area in consistently high numbers, acquired the virus through hematophagous feeding on recently infected, migrating birds. Once introduced to local mosquitoes, virus is amplified among susceptible resident birds fed upon by ornithophilic mosquitoes. This pattern allows perpetuation and subsequent establishment of virus in a continuous transmission cycle, as opposed to infection of dead-end hosts, e.g., horses. This is the first report of WNV infection in South American birds and definitive establishment of the virus in South America.

We observed varying WNV seroprevalence rates in birds and horses across regions in Venezuela (Figure). These differences reflect the focal and stochastic nature of arbovirus transmission, which depends upon many ecologic factors. One possible explanation for the greater seroprevalence in the central and eastern llanos (savannahs) and valley regions, compared with the coastal western region of Zulia State (p<0.0001, by Pearson’s χ^2^ test) would be virus introduction by migrating birds by an eastern migration route.

**Figure:**
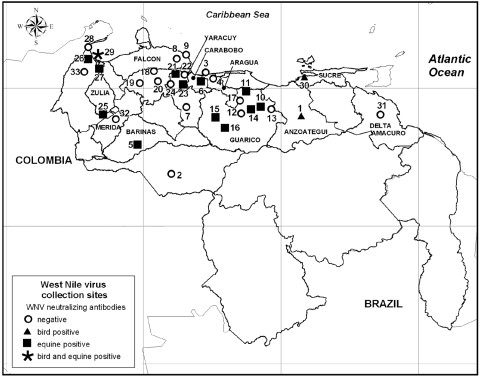
Collection sites for West Nile virus (WNV) in Venezuela. Symbols represent results of tests for specific antibodies to WNV in serum samples of birds and horses (viral titers in a 90% plaque reduction neutralization test >40 and a 4-fold differential inhibition in a neutralization assay to WNV compared with other related flaviviruses). Source: Instituto Geográfico de Venezuela Simón Bolivar, Caracas, Venezuela.

Existence of several closely related flaviviruses in the American tropics (*8*–*10*) may convey cross-protection in animals (e.g., ILHV and SLEV) or humans (dengue viruses, yellow fever virus), thereby potentially diminishing disease caused by a newly introduced flavivirus such as WNV. Although ILHV infection has not been detected in Venezuela, this flavivirus is prevalent in Brazil, Peru, French Guyana, Trinidad, and Colombia. Our study demonstrated widespread distribution of ILHV in Venezuela. Other South American flaviviruses, such as Bussuquara, Cacipacore, and Iguape, and as yet undiscovered viruses may also circulate in Venezuela.

We encourage those involved in the public and animal health systems in Venezuela to consider zoonotic flaviviruses in the differential diagnoses of human and equine cases of encephalitis and to consider ecologic surveillance for zoonotic flaviviruses in mosquito and vertebrate host populations. We recommend monitoring blood and organ donations for flavivirus infections. Our study sheds light on flavivirus distribution in Venezuela. However, nothing else is known about the ecology of zoonotic flaviviruses in this country. Such knowledge will be essential for designing effective surveillance and control should these viruses be shown to cause human illnesses.

## Supplementary Material

Appendix Table Locations in Venezuela sampled for West Nile virus
