# Supplementary material for: West Nile Virus, Venezuela
Source: Emerg Infect Dis. 2007 Apr;13(4):651–3. doi: 10.3201/eid1304.061383 (PMC2725982; doi:10.3201/eid1304.061383)
Supplement: Appendix Table — Locations in Venezuela sampled for West Nile virus [file 06-1383_appT-s1.pdf]

Online Appendix Table. Locations in Venezuela sampled for West Nile virus

ID	State	County, Location	Regional ecosystem*	No. serum samples tested (no. positive)†	
				Birds	Equines
4	Aragua	Guirardot, Portachuelos	Coast	96 (0)	
8	Falcón	Manuere, Guayabal	Coast		7 (0)
25	Zulia	Francisco J. Pulgar	Coast		6 (1)
26	Zulia	Mara	Coast		9 (1)
33	Zulia	La C. de Urdaneta	Coast	12 (0)	
27	Zulia	Maracaibo	Coast		327 (1)
28	Zulia	Paez, Goajira	Coast		6 (0)
2	Apure	Romulo Gallegos	Llanos		25 (0)
10	Guarico	Rivas	Llanos		43 (1)
11	Guarico	Monagas, Altgr. Orituco	Llanos		6 (3)
13	Guarico	Ortiz, Zaraza	Llanos		5 (0)
14	Guarico	Infante, V. de la Pascua	Llanos		18 (3)
15	Guarico	Miranda, Guarico Dam	Llanos		50 (3)
16	Guarico	Miranda	Llanos		13 (5)
21	Yaracuy	Bolivar	Valley		27 (2)
23	Yaracuy	S. Felipe, Guaquira farm	Valley	75 (0)	24 (8)
22	Yaracuy	Veroes	Valley		18 (0)
5	Barinas	Pedraza	Llanos		9 (4)
7	Cojedes	Pao	Llanos		4 (0)
12	Guarico	Las Mercedes	Llanos		3 (0)
17	Guarico	Monagas	Llanos		4 (0)
18	Lara	Urdaneta	Valley		1 (0)
19	Lara	Torres	Valley		3 (0)
20	Lara	Iribarren	Valley		3 (0)
24	Yaracuy	Sucre	Valley		1 (0)
32	Merida	Libertador, Merida	Mountain		5 (0)
3	Aragua	Girardot, Cata farm	Coast	52 (0)	1 (0)
6	Carabobo	Valencia, Lake	Valley	16 (0)	173 (2)
29	Zulia	Paez, Sinamaica Lagoon	Coast	142 (1)	
30	Sucre	Sucre, Cumana	Coast	7 (1)	
1	Anzoategui	Miranda, F. El Tigre	Llanos	56 (3)	
9	Falcón	Iturriza, Cuare Reserve	Coast	48 (0)	
31	Delta Amacuro	Tucupita	Delta system	72 (0)	
	Total			576 (5)	791 (34)

*Llanos, savannah.
†Positive by 90% plaque reduction neutralization test.
